# Macular perfusion changes in people administered two types of COVID-19 vaccines: comment

**DOI:** 10.5935/0004-2749.2024-0065

**Published:** 2024-06-20

**Authors:** Hinpetch Daungsupawong, Viroj Wiwanitkit

**Affiliations:** 1 Private Academic Consultant, Phonhong, Lao People’s Democratic Republic; 2 University Centre for Research & Development Department of Pharmaceutical Sciences, Chandigarh University, Mohali, Punjab, India

Dear Editor,

Here, we discuss the article “Macular perfusion changes in people administered two types of COVID-19 vaccines: optical coherence tomography angiography study^(1)^”. Participants in a cohort study who received two doses of the CoronaVac vaccine and one dose of the BNT162b2 vaccine underwent pre- and post-vaccination ophthalmologic tests and imaging procedures. Following immunization with both vaccinations, vascular densities in the superficial and deep capillary plexuses as well as choriocapillaris flow significantly decreased.

The study’s modest sample size-just 24 people, each of whom had one eye evaluated-is a limitation. Furthermore, there was no control group available for comparison. There were no long-term data on the effects of the vaccinations on the ocular vasculature available, and the follow-up time was brief. Future research on the long-term effects of COVID-19 vaccinations on ocular health should employ bigger sample sizes and longer follow-up times to overcome these shortcomings. It would be beneficial to compare the effects of various vaccination types on the ocular vasculature by including a control group. Additionally, studying the fundamental processes driving alterations in the ocular vasculature following immunization may provide crucial insights into how vaccinations affect eye health.

In addition to these recommendations, other influences of COVID-19 vaccinations on ocular health should be investigated: for example, examining the possible mechanism behind the noted modifications in macular perfusion after immunization. Elucidating the underlying pathophysiology may help to clarify how immunization affects ocular vasculature. Not to mention how the COVID-19 vaccine affects other eye characteristics like visual acuity, thickness of the retinal nerve fiber layer, and intraocular pressure. Studies with long follow-up periods that are longitudinal can also be beneficial.
