# Supplementary material for: Macular perfusion changes in people administered two types of COVID-19 vaccines: comment
Source: Arq Bras Oftalmol. 2024 Jun 20;87(4):e2024-0065. doi: 10.5935/0004-2749.2024-0065 (PMC11623390; doi:10.5935/0004-2749.2024-0065)
Supplement: Related texts

# Reply: Response to Daungsupawong and Wiwanitkit

**Authors:** Zeynep Serikoglu Akbas, Tuna Ozan, Ceyhun Arici

**Affiliations:** 1 Department of Ophthalmology, Cerrahpasa Medical Faculty, Istanbul University- Cerrahpasa, Istanbul, Turkey; 2 Department of Ophthalmology, Beyoglu Eye Training and Research Hospital, University of Health Sciences Turkey, Istanbul, Turkey

Dear Editors,

We welcome the response of Drs. Daungsupawong and Wiwanitkit to our article examining macular perfusion changes in patients who received two types of COVID-19 vaccines. We would like to note that the COVID-19 vaccine was administered in Turkey only to a limited group that included healthcare workers and the elderly given the health emergency at that time. Consequently, we were restricted to a relatively small sample size. We understand the necessity and importance of studying effects of these vaccines in larger groups and acknowledge this as a limitation of our study.

Our study includes a control group; however, this group was specifically designed to test the repeatability of optical coherence tomography angiography measurements. It was determined that a prospective comparison within the sample group itself would yield more objective results.

In the literature, most ocular vascular events following COVID-19 vaccinations occur within the first two weeks, rarely extending to four weeks. Consequently, we designed the follow-up period to be 2-4 weeks^(1^,^2^,^3)^.

We acknowledge and appreciate the authors’ suggestions, as conducting future studies with larger sample sizes, more parameters, longer follow-up periods, and comparison with control groups receiving other vaccines will significantly enhance our understanding of ocular health.

## References

[2] Girbardt C, Busch C, Al-Sheikh M, Gunzinger JM, Invernizzi A, Xhepa A (2021). Retinal vascular events after mRNA and adenoviral-vectored
COVID-19 vaccines-A Case Series. Vaccines (Basel [Internet].

[3] Ng XL, Betzler BK, Testi I, Ho SL, Tien M, Ngo WK (2021). Ocular adverse events after COVID-19
vaccination. Ocul Immunol Inflamm [Internet].

[4] Valenzuela DA, Groth S, Taubenslag KJ, Gangaputra S (2021). Acute macular neuroretinopathy following Pfizer-BioNTech
COVID-19 vaccination. Am J Ophthalmol Case Rep [Internet].
